# Information flow during gene activation by signaling molecules: ethylene transduction in *Arabidopsis *cells as a study system

**DOI:** 10.1186/1752-0509-3-48

**Published:** 2009-05-05

**Authors:** José Díaz, Elena R Alvarez-Buylla

**Affiliations:** 1 Facultad de Ciencias Universidad Autónoma del Estado de Morelos Cuernavaca, Morelos 62209, México; 2 Centro de Ciencias de la Complejidad Universidad Nacional Autónoma de México Cd Universitaria, México DF 04510, México; 3 Departamento de Ecología Funcional Instituto de Ecología Universidad Nacional Autónoma de México Cd Universitaria, México DF 04510, México; 4 Facultad de Ciencias, Universidad Autónoma del Estado de Morelos, Av Universidad 1001, Colonia Chamilpa, Cuernavaca 62209, México

## Abstract

**Background:**

We study root cells from the model plant *Arabidopsis thaliana *and the communication channel conformed by the ethylene signal transduction pathway. A basic equation taken from our previous work relates the probability of expression of the gene *ERF*1 to the concentration of ethylene.

**Results:**

The above equation is used to compute the Shannon entropy (*H*) or degree of uncertainty that the genetic machinery has during the decoding of the message encoded by the ethylene specific receptors embedded in the endoplasmic reticulum membrane and transmitted into the nucleus by the ethylene signaling pathway. We show that the amount of information associated with the expression of the master gene *ERF*1 (Ethylene Response Factor 1) can be computed. Then we examine the system response to sinusoidal input signals with varying frequencies to determine if the cell can distinguish between different regimes of information flow from the environment. Our results demonstrate that the amount of information managed by the root cell can be correlated with the frequency of the input signal.

**Conclusion:**

The ethylene signaling pathway cuts off very low and very high frequencies, allowing a window of frequency response in which the nucleus reads the incoming message as a sinusoidal input. Out of this window the nucleus reads the input message as an approximately non-varying one. From this frequency response analysis we estimate: a) the gain of the system during the synthesis of the protein ERF1 (~-5.6 dB); b) the rate of information transfer (0.003 bits) during the transport of each new ERF1 molecule into the nucleus and c) the time of synthesis of each new ERF1 molecule (~21.3 s). Finally, we demonstrate that in the case of the system of a single master gene (*ERF*1) and a single slave gene (*HLS*1), the total Shannon entropy is completely determined by the uncertainty associated with the expression of the master gene. A second proposition shows that the Shannon entropy associated with the expression of the *HLS*1 gene determines the information content of the system that is related to the interaction of the antagonistic genes *ARF*1, 2 and *HLS*1.

## Background

Networks of intracellular processes continuously adjust in order to trigger for specific genetic responses to the flow of environmental information received by different cell compartments [1]. For example, the plasma membrane contains proteins that function as specific receptors of various signaling molecules (hormones and growth factors) that transmit information about the environmental conditions of the cell to the nucleus via transduction pathways. Within the nucleus, regulatory transcriptional mechanisms control gene expression [2]. As sessile organisms, plants must continuously adjust their growth and development to changing environmental conditions and challenges [3]. Thus, plant signaling pathways are appropriate study systems to tackle questions concerning the complexity of such pathways and the mechanisms that enable living cells to sense, integrate, and respond to complex environmental signals.

In classical biochemistry, signaling pathways are modeled as simple relay systems in which one molecule activates a downstream molecule that in turn activates another to lead to a biochemical or genetic outcome that helps the organism respond to changes in its environment.

As the signal transduction literature grows, however, it is becoming clear that intracellular signaling pathways are interconnected rather than linear and that they form complex networks that process information from the environment before such information reaches the nucleus. Thus signals induce changes in both cytoplasmic reactions and in the expression of the cell's genetic machinery [4]. For example, in the *Xenopus *blastomeres, the MAPK cascade cross talks with the calcium signaling system [5]. In fibroblast cells, the EGF and FGF signaling systems share the same MAPK signaling cascade in order to transmit their information to the nucleus [6]. In *Saccharomyces cerevisiae*, the Ras-cAMP signaling system is immersed in a dense network of signaling molecules [7].

Ethylene is a phytohormone that activates defense responses to infections and to several types of stress in plants [8,9]. In *Arabidopsis *root cells, specific ethylene receptors are located in the endoplasmic reticulum (ER) rather than in the plasma membrane. These receptors (ETR1, ETR2, ERS1, ERS2, EIN4) are ER membrane proteins that form a dimeric unit [10,11].

In the absence of ethylene, the dimeric unit is in its active state. Its kinase domain activates a downstream Raf-like protein, the CTR1 kinase, which inactivates the EIN2 protein through a MAPK-like cascade. When ethylene binds to either of the specific sets of receptors, it inactivates the CTR1 cascade, allowing activation of the EIN2 protein. The localization of this molecule in the ER – nuclear membrane complex has not still been resolved but EIN2 turns on the transcription factor EIN3 inside the nucleus. This transcription factor binds to the promoter of *ERF*1, which triggers the so-called "triple response" of etiolated seedlings and defense mechanisms that depend on ethylene signaling [9,10]. Thus, the dynamics of the *Arabidopsis *root cell response mechanism to ethylene are based on a two-module structure that can switch from one module to the other, depending on the presence of ethylene in the environment of the root cell [12].

### The communication channel

A typical communication channel consists of a source, an encoder, a noisy channel, a decoder, and an effect [13]. In this case, the source is ethylene, the encoder is the ethylene receptor, and the noisy channel consists of the molecular machinery associated with the ethylene response. The decoder is the master gene *ERF*1, and the effect is the gene's response to ethylene.

In the system being modeled here, the message that the cell receives from its environment (i.e., the concentration of the ethylene phytohormone) is encoded in the number of ethylene specific receptors that are *inactivated *at a given time. In the case of a single cell, the perception of the signal may be independent of the spatial distribution of the receptors if a uniformly distributed signal is assumed [2]. This is the case in the model presented here.

Once ethylene activates the signal transduction pathway, this signaling system transfers information to the nucleus, where specific genes are transcribed in response to the ethylene signal. Once the signal has been encoded, it has to be transmitted to the nucleus through a noisy channel (*noise *is a general term for anything that tends to produce errors in transmission). This channel consists of the CTR1-MAPK module and its negative effect on the EIN2 molecule. The message carried by the ethylene concentration should be transmitted with fidelity to the nucleus, i.e. the amount of EIN3 activated molecules should be proportional to the intensity of the signal, which is measured by the proportion of inactivated ETRs. In the nucleus, the activation of the *ERF*1 genetic machinery depends completely on inactivation of the CTR1 molecule [12].

In this work, we have been able to address two key issues in this and other signal transduction processes. First, we propose a novel approach for measuring the information content of a given message elicited by a given concentration of an agonist molecule (ethylene in this case), and which triggers a specific genetic response (Table 1). In the system analyzed in the present study, the message is sent from the ER surface to the nucleus via the ethylene communication channel. Second, we propose a means by which cells elicit a particular genetic response depending on the information content in the message delivered by an agonist molecule (ethylene in this case). We achieve this by relying on the relationship between the amount of ethylene applied to the root cell and the probability of expression of the *ERF*1 gene (Table 1) [12]. Once the probability distribution for the *ERF*1-dependent expression of a series of genes as a response to the agonist concentration is established, the amount of uncertainty in the content of the message dispatched from the receptors of the cell membrane (or from the ER surface, in this case) can be readily measured using Shannon's entropy, *H*. Using *H*, we can calculate the amount of information in the message carried by the ethylene signaling pathway into the nucleus [12,14,15].

**Table 1:** and *I *(*mers*) as a function of ethylene concentration *ET *(*μL/L*) for the root cell of *Arabidopsis*. The values for  and *I *were calculated using equations (1) and (5).

***ET*** **(*μ*L/L)**		***I *= *ln*(2) - *H*** **(*mers*)**
0.0001	0.0002843	0.697

0.0005	0.00135	0.689

0.001	0.00264	0.681

0.005	0.012	0.634

0.01	0.024	0.586

0.05	0.105	0.364

0.1	0.1874	0.217

0.5	0.522	0.007

1	0.679	0.072

5	0.905	0.386

10	0.946	0.489

15	0.96	0.532

50	0.982	0.609

Thus, we propose using Shannon's entropy of the gene expression profile of a root cell exposed to ethylene in order to explore the information content of the messages elicited by this phytohormone and sent from the ER surface to the nucleus, where gene regulation takes place. We derive the model proposed here from that in [12] (*see also *Additional file 1) in order to calculate the amount of information that the root cell obtains from its environment during *ERF*1 activation for a given ethylene concentration. This approach is used to evaluate how the cell may translate a *specific gene activation probabilistic distribution*, elicited in response to a given concentration of a signaling molecule (a phytohormone in this case), into an *information value*. The approach proposed here can be applied to any other signal transduction pathway.

## Methods

### Root cell modeled as a three-compartment system

The ethylene receptor, which appears to be located in the membrane of the endoplasmic reticulum (ER) of the root cell, induces a chemical reaction inside the ER lumen, which has a volume *V*_*ER*_. This volume, defined as the first compartment in the model, can be used to model the concentration of all the signaling molecules of the MAPK cascade. Inactivation of the ethylene receptors activates a series of transcriptional processes in the nucleus of the root cell, which can be considered the second compartment, with volume *V*_*nucleus*_. Both compartments are enclosed in a rectangular cylindrical cell with a diameter of 30 *μ*m and a height of 10 *μ*m, and this space can be taken as the third compartment. We assume that the reactions of the MAPK module occur inside the ER main body, which is modeled as a cylinder with a 1 *μ*m diameter and 10 *μ*m length [16]. Consequently, *V*_*ER *_is approximately 7.86 *μ*m^3 ^(7.86 × 10^-15 ^L) in our model.

The nucleus can be modeled as a sphere with a diameter of 10 *μ*m, which implies that *V*_*nucleus *_is 524 *μ*m^3 ^(5.24 × 10^-13^L). The concentrations of the molecules that are transported in either direction between the ER and nucleus can now be described by the ratio *V*_*nucleus*_/*V*_*ER*_, which, based on our values, is 66.5. Assuming that the concentration of a molecule *k *in the ER at time *t *is *c*_*k*_(*t*), then if this molecule moved into the nucleus, either by diffusion or by transport of any kind, its concentration would be: *C*_*k*_(*t*) = *c*_*k*_(*t*)*V*_*ER*_/*V*_*nucleus*_. Thus, the concentration of a molecule *k *in the ER is 0.015-fold lower than in the nucleus. Likewise, for movements in the opposite direction, the concentration of a molecule *k *is 66.6-fold higher with respect to its concentration in the ER.

### The full model for activation of the *ERF1 *gene activation by ethylene

In Additional file 2, we present only a brief summary of the model of the activation of the *ERF1 *gene as a function of its repressor CTR1 and two families of receptors under the action of the phytohormone ethylene. In Additional file 1, we present a more detailed account of the model. A full version of the model can be found in [12], and it is based primarily on experimental results from [17]. The full version of the model is solved using the Corrector-Predictor Euler Method with a fixed time step of 0.04 s.

## Results

### I. Information flow in root cells

In an earlier paper [12], we postulated a model that links the probability of *ERF*1 expression to ethylene availability (*ET *measured in *μ*L/L) in root cells:

(1)

This model is able to reproduce some aspects of the root cell response to ethylene that had been experimentally documented by [17] if it is assumed that *ERF*1 acts as a master regulator of the ethylene cell response [18].

In equation (1), the probability of *ERF*1 being "on" and expressed depends on the agonist concentration, with a continuous or graded response to different doses of ethylene. Ethylene activates genes such as *PDF*1 [19] and *HLS*1 [20]. *HLS*1 in turn blocks the activity of genes such as *ARF*1 and *ARF*2, thus mediating the interaction of the ethylene and auxin response pathways in root cells [20,21]. The expression of *ERF*1 affects *n *genes, thus defining a multidimensional probability space describing the expression of genes in response to ethylene [12].

To account for the way in which the cell senses, integrates, and responds to the environmental information that triggers the ethylene or other signal transduction pathways, we use the Shannon's entropy function *H *[14]:

(2)

where *p*_*j *_is the probability of a given event in a set of *j *= 1, 2,..., *n *events.

In the case of a gene that can alternate between "*on*" and "*off*" expression states, the *H *function can be rewritten as:

(3)

where *p*_*j *_is the probability of the gene's state *j *at time *t *and where *H *is measured in "*mers*" because we use natural logarithms instead of base-2 logarithms or bits [14]. In the ethylene-gene response model, *p*_*j*_*(t) *is calculated from a Markov model [[12], *see *Additional file 1].

Although the *H *function is incorrectly referred to as the "amount of information" of a system, the flow of information through a communication channel can easily be measured using the *I *function [14], which is defined as:

(4)

where *H*_*max *_is the maximum value of Shannon's *H *function [14]. In other words, the information content of a message is the difference between the maximum amount of Shannon entropy minus the entropy at a given time *t*.

Thus, for a single gene, the *I *function is given by:

(5)

*H *is a function of the probability of each gene's *expression *state ("*on*" or "*off*"), and the probability of each gene's *expression *state is a function of time. Thus, *I *is a function of *H *and it also depends on the probability of each gene's *expression *state and time.

### II. Information and Entropy in gene arrays

#### Case I: One Gene

**Figure 1:**
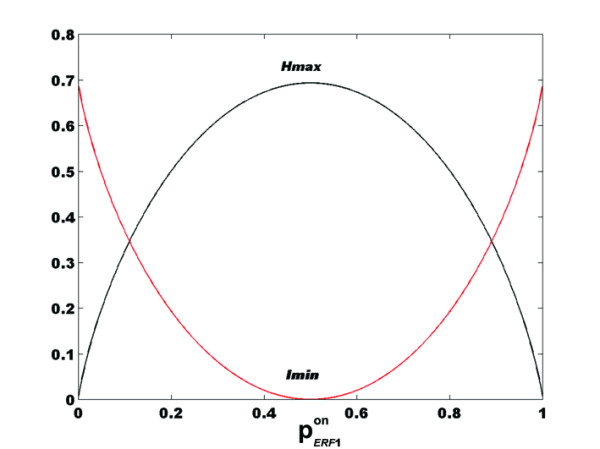
**Dependence of *I*_*ERF*1 _(black line) and *H*_*ERF*1 _(red line) functions with **. This figure illustrates the dependence of *I*_*ERF*1 _and *H*_*ERF*1 _functions with increasing probability that the *ERF*1 gene is in the "*on" *state. As expected, the minimum value of *I*_*ERF*1 _corresponds to the maximum value of *H*_*ERF*1 _when  = 0.5

*ERF*1 is activated by ethylene in a dose-dependent fashion that can be modeled by the probability of expression  for any given ethylene concentration [12]. Thus, the Shannon's *H *function for this gene is given by:

(6)

which leads to:

(7)

where:

(8)

which corresponds to the maximum uncertainty in the value of  = 0.5 (Figure 1).

From equations (4), (5), (7) and (8), we obtain:

(9)

As expected, the *I*_*ERF*1 _function decreases as the *H*_*ERF*1 _function increases when  increases from 0 to 0.5. *H*_*ERF*1 _reaches its maximum value and *I*_*ERF*1 _is minimum when  = 0.5 (see Table 1).

**Figure 2:**
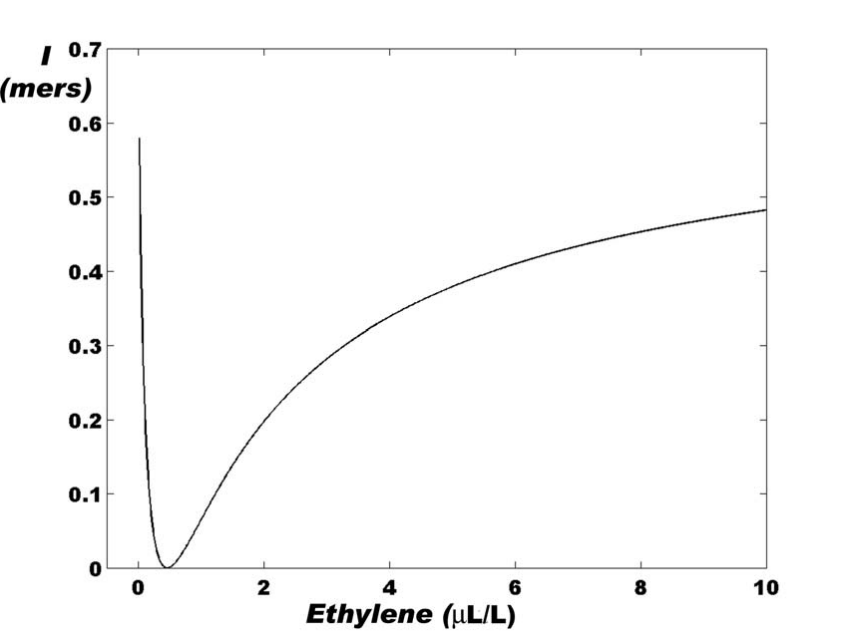
**Relationship between the *I*_*ERF*1 _function (*mers*) and the ethylene concentration (*μ*L/L) in the root cells of *Arabidopsis thaliana***. An asymmetric potential-like curve is clearly observed, with a minimum located at an ethylene concentration of ~0.5 *μ*L/L. The curve was calculated using equations (1) and (9). Also see Table 1.

According to equation (1), we can calculate  as a function of ethylene concentration (*μ*L/L) and, in turn, calculate the value of *I*_*ERF*1 _for each value of ethylene concentration. In Figure 2, we show the corresponding graph of this relationship, and a potential-like curve is observed. The minimum value of the *I*_*ERF*1 _function is reached at an ethylene concentration of ~0.5 *μ*L/L [14]. At this ethylene concentration, CTR1* is still activated in the ER of the root cell, while the *ERF*1 gene has a probability of expression of ~0.5 [12].

**Figure 3:**
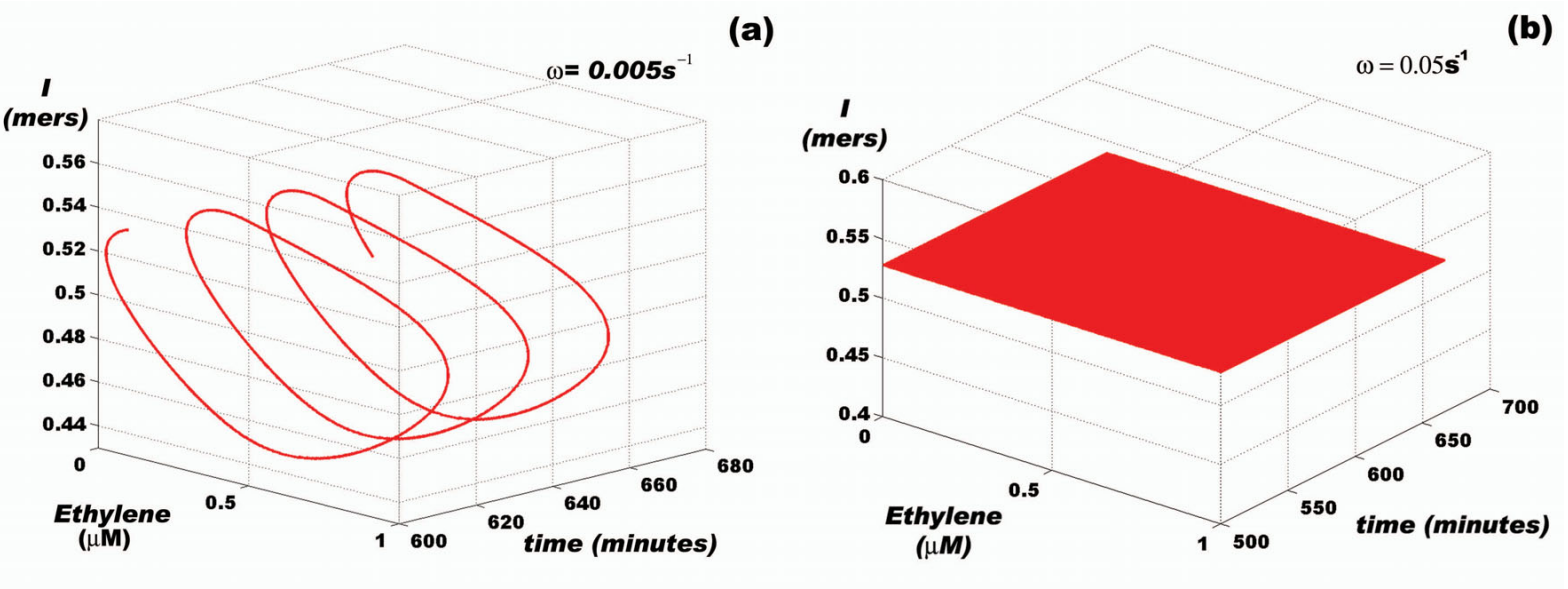
**Flow of information in the root cell as a function of the angular frequency of ethylene oscillations**. (a) Ethylene input at low frequency, (b) Ethylene input at high frequency. The process of *ERF*1 activation can clearly discriminate between the different modes of ethylene action. Both panels were calculated using the full version of the model presented in [12] (see Table 2 and Additional file 1).

**Figure 4:**
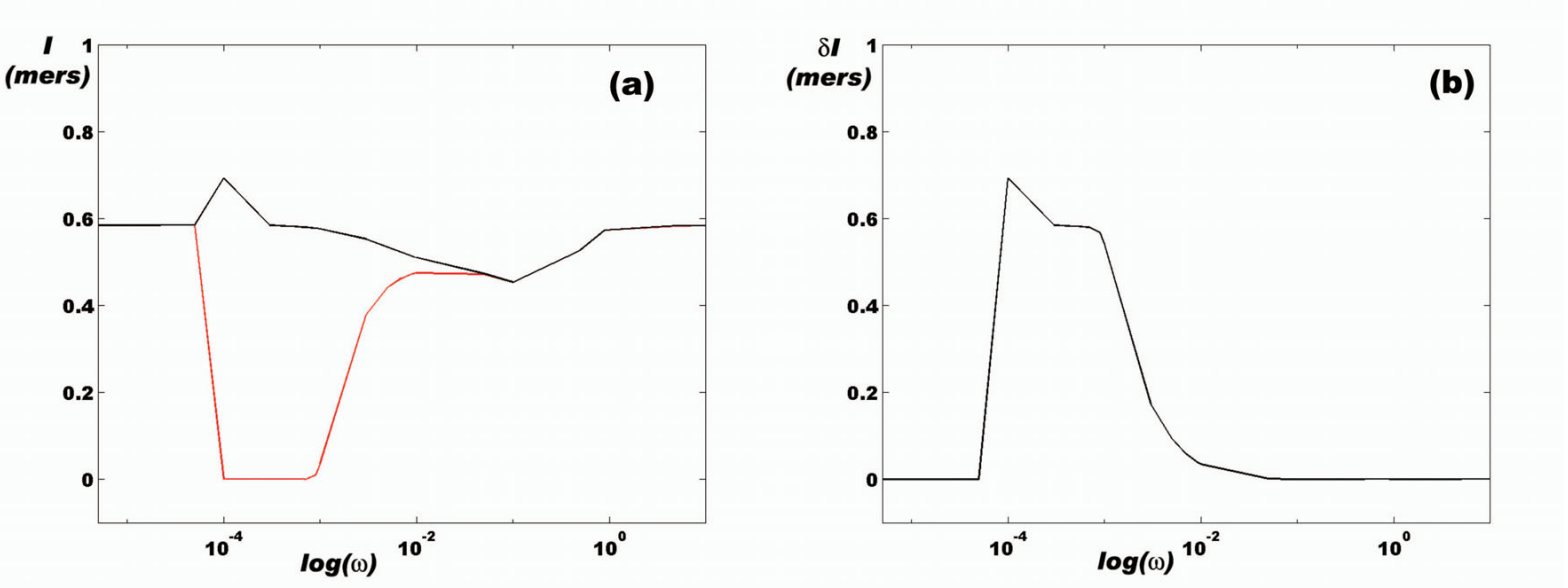
**Flow of information in the root cell as a function of the logarithm of the angular frequency of ethylene oscillations**. (a) In this panel, a window of maximum and minimum information value is shown, indicating that a set of *ω *values exists for which *ERF*1 continuously transits between its *on *state and its *off *state, i.e. the CTR1 and EIN2 molecules are alternately activated. (b) *δI *= *I*_*max *_-*I*_*min *_graph corresponding to panel (a).

In order to test this model, we explored the behavior of *I *in response to periodic variations of ethylene concentrations for different angular frequencies (*ω*). We found that the amount of information that the communication channel manages depends on *ω *and that the system can clearly discern among different regimes of input information flow. Figures 3a and 4a show that for *ω *values between 5 × 10^-5 ^and 10^-1 ^s^-1 ^(periods between 1 minute and 34 hours), the amount of information fluctuates between a minimum and a maximum value. *δI *= *I*_*max *_- *I *indicates the changes in the amount of information that the cell senses as the concentration of ethylene changes. As expected, the maximum value of *δI *is obtained precisely in the interval of *ω *values for which  switches between its minimum and maximum values [12] (Figures 4a and 4b).

**Figure 5:**
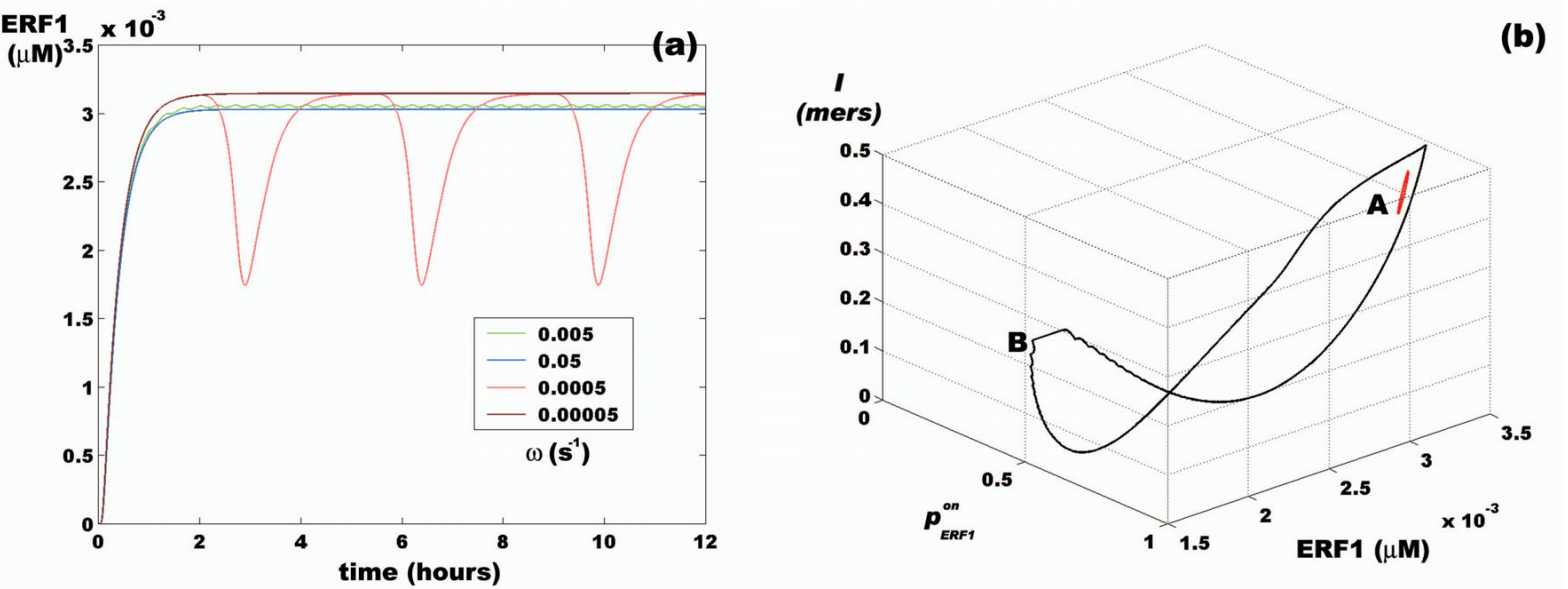
**Systems response to sinusoidal ethylene input**. (a) Amount of ERF1 accumulated in the root cell nucleus for different values of *ω *of a periodic variation of ethylene according to the function *ET = 0.5 + 0.5sin (ωt)*. The values of *ω *are shown in the small square inside the figure. (b) Amount of information managed by the ethylene root cell as a function of the probability of expression of the *ERF*1 gene and the amount of ERF1 protein in the nucleus. **A **denotes a *ω *value of 0.005 s^-1^; **B**, a value of 0.0005 s^-1^.

As pointed out in [12], the two-module structure of the ethylene response pathway has filtering properties with respect to the genetic machinery downstream of *ERF*1. However, our model predicts that levels of ERF1 protein inside the nucleus should reflect the periodic variations in the ethylene concentration. This can be tested experimentally. As shown in Figure 5a, although the probability of expression of *ERF*1 oscillates at *ω *= 0.005 s^-1^, these small-amplitude oscillations cause insignificant changes in the nuclear concentration of this protein [12]. However, ERF1 protein oscillations are more evident for *ω *close to 0.0005 s^-1 ^(equivalent to a period *T *≈ 3.5 h). Figure 5b shows the amount of information that the cell communication channel manages as a function of the probability of expression of *ERF*1, as well as the amount of ERF1 protein inside the nucleus.

As expected, the amplitude of the cycle in the 3D phase space for *ω *= 0.0005 s^-1 ^is wider than for the *ω *= 0.005 s^-1 ^case. In the first case, the amount of information flowing through the communication channel changes from ~0, when the probability of expression of the *ERF*1 gene () is close to 0.5 and the amount of the ERF1 protein inside the nucleus is ~1.7 nM, to a value ~0.5 mers when  ≈ 1 and the amount of ERF1 protein inside the nucleus is ~3 nM. These results indicate that *the amount of information managed by the root cell can correlate with the amount of a specific protein synthesized by the system at a given time*.

### Case II: One gene that is positively regulated by *ERF*1

In this case, the gene *ERF*1 turns on the gene *HLS*1 (HOOKLESS1) [8]. Thus, if we define the following events:

"*hls1*: state of expression of the gene *HLS*1 *"*

and

"*erf1*: state of expression of the gene *ERF*1*"*

the information that the gene *HLS*1 has about the state of expression of the master gene *ERF*1 is given by:

(10)

where *H*(*erf*1|*hls*1) is the entropy of *the *event *erf1 *conditional on the event *hls1*, and represents the entropy in the state of expression of the master gene *ERF1 *if the state of expression of the slave gene *HLS*1 is known. *H*(*erf*1|*hls*1) can be calculated by the expression:

(11)

where *H*(*ERF*1|*HLS*1^(*i*)^) is defined by the expression:

(12)

For equation (12) we have the following possibilities: if we assign *i *= *j *= 1 to the "*on*" or expressed state and *i *= *j *= 2 to the "*off *" or not expressed state of the respective genes, we have *p*(*ERF*1^(*j*) ^∩ *HLS*1^(*i*)^) = 0 when *j *≠ *i*, and *p*(*ERF*1^(*j*)^|*HLS*1^(*i*)^) ≈ 1, when *i *= *j*.

Hence, in the absence of other genes that could activate *HLS*1 [8], from (11) and (12) we get:

(13)

Therefore, from equation (10) we finally state that:

(14)

Consequently, the expression of *HLS*1 is completely defined by the expression of *ERF*1. In turn, equation (14) implies that the information managed by the system when both genes interact is completely determined by the Shannon's entropy associated with the expression of the master gene *ERF*1.

### Case III: One gene that is negatively regulated by *HLS*1

In this case, the activation of *HLS*1 induces the inactivation of the *ARF*2 gene [8]. Thus, when *HLS*1 is in the "*on*" state at time *t *with probability , *ARF*2 is in the "*off*" state with probability  at time *t*. Defining the events:

"*hls1*: state of activation of the gene *HLS*1 *"*

and

"*arf2*: state of activation of the gene *ARF*2*"*

we can write:

(15)

where the "*on*" state is denoted by 1 and the "*off *" state by 2 for each gene. Thus, we have:

(16)

For equation (16) we have the following possibilities:

We know that:

(17)

where *p*(*HLS*1^(*on*)^|*ARF*2^(*on*)^) = 0 and *p*(*HLS*1^(*on*)^|*ARF*2^(*off*)^) = 1. If *Ω *is the whole space of events associated with these two genes, whose expression is mutually exclusive but not independent, then it is clear that *ARF*1^*off *^∩ *HLS*1^(*on*) ^= *HLS*1^(*on*) ^and .

If we assign *i *= *j *= 1 to the "*on*" or expressed state and *i *= *j *= 2 to the "*off *" or not expressed state of the respective genes, we have *p*(*ARF*1^(*i*) ^∩ *HLS*1^(*j*)^) = 0 when *j *= *i*,, and *p*(*HLS*1^(*j*)^|*ARF*2^(*i*)^) ≈ 1, when *i *≠ *j*.

Hence, in the absence of other genes that could activate *HLS*1 [8], from (15) and (16) we get:

(18)

Therefore, we obtain:

(19)

Equation (19) can be computed from the full version of the model (see model section). We can use this equation to estimate the amount of Shannon entropy from the auxin communication channel that is managed by the ethylene communication channel due to the repression of *HLS*1 over *ARF*2. According to equation (19), this amount is equal to the Shannon entropy associated with the expression of the *HLS*1 gene alone. This amount of Shannon entropy, in turn, is less than or equal to the Shannon entropy associated with the expression of its master gene *ERF*1 (see case II above). As usual, the amount of information interchanged by these two channels is given by equation (4):

(20)

In this case, *H*(*arf*2|*hls*1)_*MAX *_= ln2 because both communication channels possess two inaccessible states.

## Discussion

### Amount of information carried by a message from the phytohormone receptor to a gene effector

In contrast to the classical views of signaling pathways as simple relay systems, biochemical and cell biological experiments indicate that intracellular signaling mechanisms involve dense networks of interacting molecules in which information from the cell environment is processed before it reaches the nucleus [4]. An *information theory *approach can help us understand how this incoming message from the cell or ER surface is processed and transmitted into the nucleus under intracellular conditions in which numerous proteins interact.

In this paper, we have presented a novel approach to understanding how information is managed in the ethylene signal transduction pathway, which is fundamental for plant responses to environmental cues. In the present case, the transfer of information from the membrane to the nucleus is indirect because the response is based on the inactivation of CTR1 and downstream molecules [12]. In such system, we have been able to address the question of how much information the communication channel can manage. We have achieved this by calculating the probability of *ERF*1 gene expression for a given amount of ethylene applied to the root cell, and using this result to determine how much information the ethylene-*ERF*1 system handles at a given time [12]. Our implementation (Eq. 1) let us use the Shannon entropy definition (Eq. 3) to determine the uncertainty associated with the flow of information through this communication channel, from the ER-embedded ethylene receptor to the *ERF*1 gene in the nucleus. We then used equation (5) to calculate the amount of information that is associated with the activation of *ERF*1.

According to Figure 1, when the probability of expression of *ERF*1 is 0, the cell has a minimum Shannon entropy and a maximum amount of information from its environment because the CTR1 module is switched on and the EIN3 module is switched off. The root features dependent on auxin are fully expressed, and the *ARF*2 gene is expressed. As the ethylene concentration increases, the probability of expression of *ERF*1 increases, but the amount of information decreases because the fraction of activated EIN3 molecules is insufficient to completely counterbalance the effects of the CTR1 module, and the auxin response is reduced but not eliminated.

When  = 0.5, half of the auxin-dependent characteristics have been disabled, but the full ethylene response has not been expressed yet. At this point, the system manages the minimum information value and the maximum Shannon entropy or uncertainty value. This situation corresponds to cases in which the system must discern between two possibilities but does not have sufficient information to make a decision. This may correspond to a bifurcation point in the phase space where the system is equally like to take one pathway or another.

For ethylene concentrations above ~1 *μ*L/L,  is greater than 0.5 and the phenotypic characteristics associated with the triple response of etiolated seeds gradually dominant the auxin-dependent characteristics. Over 10 *μ*L/L, the ethylene-dependent communication system manages the maximum amount of incoming information from the external cell environment (~0.5 mers) and exhibits the full response to ethylene.

Figure 2 shows that this behavior of the communication channel leads to a potential-like curve when the sigmoid dose-response graph [12] is replaced with a dose-information graph. This last curve is symmetric near its minimum value and it becomes extremely asymmetric as the ethylene dose increases or decreases. Thus, as the ethylene concentration increases, the rate of information per unit of ethylene concentration rapidly falls until the minimum is reached and then rapidly increases until a maximum value is attained. At least until 10 *μ*L/L, however, the amount of information that the ethylene-dependent communication channel carries is always less than the information that the channel carries in the absence of ethylene. This may be due to the fact that the effect of ethylene requires the prior inactivation of the ETR and the indirect activation of the *ERF*1 genetic machinery.

From equation (1) we have , and from the definition of the *I*_*ERF*1 _[Equation (9)] we get:

(28)

Thus, in mathematical terms, the characteristics of the curve in Figure 2 can be written as:  and  at *ET *~0.5 *μ*L/L. Equation (28) indicates that for the first time, *we can measure the amount of information that a given hormone carries into a genetic communication channel and that this dependence is non-linear and follows a potential-like curve*.

In summary, we have shown that our approach allows us to evaluate, in several different ways, how a cellular communication channel can manage its information flow. First, we explored the amount of information released into the system by different concentrations of an agonist that are received at the ER or cell surface. It is possible that a given concentration of agonist conveys a given message involving a specific amount of information, up to the saturation of the receptor. Second, we explored how much of the total amount of information released by the agonist reaches the nucleus. This amount represents the real capacity of the channel to transmit information from the encoder with fidelity. It is possible that cells use mechanisms such as amplification, redundancy, and splitting of the message to ensure that all of the contents of the message reach the nucleus. We were also able to determine the effector's response to the information in the message transduced from the membrane. The effector should read the correct message in order to induce the correct output. The effect of *noise *(which is a general term for anything that tends to produce errors in transmission) should be minimized as much as possible in order to avoid mistakes while reading and translating the perceived messages. Thus there should be molecular mechanisms that ensure that the message sent from the receptor is interpreted correctly in the nucleus. Finally, if a message is sent from a surface receptor, there should be a code to translate it into a genetic response. We know how the genetic code is translated into a specific protein. However, we do not know how cells encode information from the activation or inactivation of surface receptors into an appropriate gene expression profile via signal transduction pathways. This encoding mechanism explains how genotypically identical cells behave differently in different environments. In this paper, we propose a novel approach to investigate this.

### The possible code used by the ethylene communication channel

If we assume that there are *N *specific ethylene receptors embedded in the ER membrane, and we denote the maximum activation level of each receptor under steady state conditions by 1 and the inactivated state by 0. Then when the occupancy level of the ethylene receptors is 0%, we have the *N*-length code , which corresponds to the outcome  in the probabilistic space for the gene expression. When the ethylene concentration is above 10 *μ*L/L, the level of activation of the receptors is ~0 [12], so that the code , with *M *≃ *N*, corresponds to the outcome  in the probabilistic space for the gene expression. In both cases, *H*_*ERF*1 _= 0 as expected.

The fraction *f *of inactivated receptors () is given by the steady-state solution of the differential equation at a given concentration of ethylene (*ET*) (see Additional file 2 and [12]):

(29)

where *etr*^*(-) *^is the concentration of ethylene-bound receptors, *etr*_*T *_is the total concentration of ethylene-specific receptors in the ER membrane,  is the dissociation constant of the receptor, and *ET *is the concentration of free ethylene.

The average *k*_*d *_value used in [12] is 6 × 10^-5 ^*μ*M = 0.00148 *μ*L/L. The reported *k*_*d *_= 0.036 *μ*L/L [22] for the ETR1 receptor in transgenic yeast expressing the *ETR*1 gene. The apparent dissociation constant for the hypocotyl-growth response reported by [17] is ~0.11 *μ*L/L. According to [11], the *k*_*d *_values of ETR families 1 and 2 are very similar.

With the more precise value of *k*_*d *_= 0.036 *μ*L/L, *R*_*T *_≈ 0.3 *μ*M with respect to the ER volume [12], and, if we assume that only receptors of ETR families 1 and 2 are present, then the fraction of *inactive *receptors in the presence of ~1 *μ*L/L of ethylene is *f *≈ 0.07 or 7%. Thus, when  = 0.5, the possible input code consists of *1-f *= 0.93*N *or 93% of active receptors and *f *= 0.07*N *or 7% of inactive receptors. Thus, we have the *N*-length code of the generic form: . For example, if *N *= 100 and we assume that the order of the 1's and 0's in the code is important, there are  possible codes compatible with the outcome  in the probabilistic gene expression space. If the order is not important, i.e. the system responds only to the temporal aspect of the signal, we have only one code. In this case, *H*_*ERF*1 _attains its maximum value (see Figure 1).

In this case, when the communication channel responds only to the temporal aspects of the external signal, there can be a one-to-one relationship between the proportion of inactivated receptors (i.e., the intensity of the signal) and the outcome in the probabilistic gene expression space: .

As we mentioned before, once the signal has been encoded it has to be transmitted to the nucleus through a noisy channel. This channel consists of the CTR1-MAPK module and its negative effect on the EIN2 molecule. The message carried by the ethylene concentration should be transmitted with fidelity to the nucleus, i.e. the amount of EIN3 activated molecules should be proportional to the intensity of the signal, which is measured by the proportion of inactivated ETRs.

### Information flow in response to a sinusoidal hormonal input

The cell's internal noise consists of all the processes that could alter the transmission and content of information of the signal from the agonist receptor to its target through a given signalling pathway. If we assume an internal noise level value of *ξ*, then the message will be reproduced with fidelity 1-*ξ*. Another interesting question arises at this point: *how does the system ensure the fidelity of the signal in a noisy environment*? One possible answer arises from the chemical structure of the communication channel: the particular combination of rate constants and concentration of signaling molecules will have the necessary noise-filtering properties for the communication channel [23]. In a previous paper, we used *in silico *experiments on the frequency distribution response to show that the filtering properties of the *ERF*1 communication channel are able to eliminate extremely low and extremely high noise frequencies, which can alter events downstream of the *ERF*1 gene [12].

Plants secrete ethylene in a nearly circadian cycle, with the maximum level of ethylene released during the day and the minimum level at night. In [12], we performed a series of *in silico *experiments in which we varied the frequency of a sinusoidal input of ethylene to explore how the system responds to periodic rhythms with contrasting frequencies. In this work, we repeated these experiments to learn how the system reads an incoming message from the environment consisting of variations in the frequency of an ethylene input signal (see Figure 3). Thus, while a slower frequency signal is read as an oscillating flow of information (Figure 3a), high frequency inputs are translated into a message with an approximately constant amount of information. Furthermore, there is a window of frequency inputs for which a message from the outside contains the maximum amount of information. Figure 4 shows that this frequency window exhibits a zero information state followed by the maximum information state, coinciding with the natural circadian behavior. *Although it is difficult to find a natural phenomena that follows an exact sinusoidal pattern of intensity fluctuations, the *in silico *experiment shown here suggests that circadian rhythms can transiently cut off the information flow from a particular communication channel (a signaling pathway) while opening the information flow from an alternative communication channel*. This switch between two alternative information flow regimes can depend, as we pointed out before, on the structural features of each signaling pathway. In the case analyzed here, the balance between the values of *k*_*on *_and *k*_*off *_for the activation of the ETR1/2 family of receptors can determine the amplitude of the maximum frequency response window of the ethylene-signaling pathway.

In this frequency response window, the gain of the system (*G*), which is measured by the log_10 _of the amplitude of the outcome signal (the amplitude of the oscillations in the concentration of ERF1 protein in the nucleus) with respect to the amplitude of the incoming signal (the amplitude of the sinusoidal wave of ethylene) [Figure 5a], tends to -∞ at an angular frequency of *ω *= 0.005 s^-1^. In contrast, the value is -5.59 dB at an angular frequency of *ω *= 0.0005 s^-1^. This means that the machinery of protein synthesis can effectively reduce the amplitude of the oscillations up to ~60 times while maintaining the frequency of the input signal; in other words, the response is *linear under steady-state conditions*.

As shown in Figure 5b, a circle in 3D space can represent this peculiar behavior of this signaling pathway. The three axes in this space represent the main features of the communication channel for two different values of *ω*: the flow of information, the probability of expression of the *ERF*1 gene, and the amount of ERF1 protein accumulated in the nucleus as a result of *ERF1 *expression. In this representation, it becomes clear that the system distinguishes between the two oscillation regimes of the incoming signal, thus giving rise to two different forms of the output signal, each with different information.

From Figure 5b, it is also clear that when the oscillating input signal has an angular frequency ~0.0005 s^-1^, the time between the minimum and the maximum values of the circle can be used to estimate the time needed for the protein synthesis machinery to recover from a ~50% decrease in its activity. The amplitude of the peak is ~1.3 nM and the recovery time is approximately 2.5 h, so that in 9000 s, an expected total of ~423 ERF1 molecules are produced assuming that the nuclear volume is on the order of 540 *μ*m^3 ^[12]. This implies that the rate of protein synthesis is on the order of ~0.047 molecules/s; in other words, each ERF1 molecule is synthesized and returned to the nucleus in ~21.3 s.

During this recovery time, the amount of information increases by ~0.5 mers, which means that each new molecule of ERF1 protein carries 0.0018 *mers *[0.0026 bits ≈ 2.6 millibits (mb)] of information into the nucleus at a rate of 8.45 × 10^-5 ^mers/s (1.22 × 10^-4^bits/s ≈ 0.1 mb/s) in the presence of periodic ethylene stimulation with *ω *= 0.0005 s^-1^. In this form, *the steady linear properties of the communication channel can be used to estimate the amount of information transferred into the nucleus for each new molecule of protein synthesized*. In addition, once it becomes possible to measure these rates within single cells, the predictions of the model presented here may be tested experimentally and used to improve the model.

### Interaction of the *ERF1 *gene with downstream genes

From the results section, the event *hls*1^*on *^⊂ *erf*1^*on *^implies that *H*_*HLS*1 _≤ *H*_*ERF*1 _because *I*(*erf*1; *hls*1) = *H*_*ERF*1_. This result means that in the case of one dependent gene, the total Shannon entropy in the communication channel is completely determined by the Shannon entropy associated with the expression of the master gene. We can express this statement as a mathematical proposition:

### Proposition 1

Define the events *hls1*^*on *^as when the gene *HLS*1 is in its expressed state due to the expression of the *ERF*1 gene, and *erf1*^*on *^as when the master gene *ERF*1 is in its expressed state, i.e. *hls*1^*on *^⊂ *erf*1^*on*^. Then:



Corollary



### Proposition 2

Define the events *hls1*^*on *^as when the gene *HLS*1 is in its expressed state due to the expression of the *ERF*1 gene and *arf2*^*on *^as when the gene *ARF*2 is in its expressed state. If these events are such that *hls*1^*on *^∩ *arf*2^*on *^= ∅ and  then:



The arguments that provide support for these propositions are found in the results section. The propositions put forward here are extremely important for understanding how the ethylene communication channel is built. The hierarchical structure of the channel is revealed when we use a probabilistic description of the genetic expression of the system instead of a deterministic one. By defining the degree of expression of the genes considered in the simulated system as a probability, we introduce a certain degree of uncertainty that can be measured using the Shannon entropy function.

We postulate that the decoder of the information carried by the ethylene concentration is the master gene *ERF*1 and thus, that *the entropy associated with the decoding of environmental information is upper bounded by the value of H for this gene*. This information decoding process causes a given number of ERF1 protein molecules to attach to the promoter sites of target genes with a CCG box and thereby trigger the ethylene response.

In this form, Proposition 1 and its corollary state that the uncertainty introduced in the communication channel by translation of the gene *HLS*1, which is expressed after *ERF*1, is due entirely to the decoding of the incoming message by *ERF*1. This proposition also implies that the translation of *HLS*1 cannot increase the level of uncertainty within the communication channel. In other words, the expression of a "slave" or dependent gene cannot produce a greater degree of uncertainty than the produced by the expression of its master gene when the incoming message created by a given hormone concentration is decoded.

Proposition 2 states that the mutually exclusive expression of the two antagonist genes *HLS*1 and *ARF*2 does not produce more entropy than that produced during the expression of either of their master genes. Although these propositions are inspired by limited and preliminary results and are applicable at this point only to the ethylene communication channel, they provide novel guides for studies of other signaling pathways in the future. They suggest that master genes may be responsible for the precise decoding of messages from the cell environment in order to guarantee certain precise responses to a signal even in noisy environments.

## Conclusion

1) Modeling of gene expression with a stochastic approach allow us to use the information theory to understand how cells use their signal transduction pathways to transmit information with fidelity from a specific receptor for an agonist to the nucleus, where this information is used to perform the adequate genetic response.

2) However, this stochastic approach suggests that we cannot determine the precise genetic response elicited by a cell under a given hormonal concentration. This amount of uncertainty in the expression of a set of genes under the action of a hormonal input reflects the effect of noise during the transmission of the message from the encoder (the specific hormonal receptor).

3) We can use the Shannon entropy (*H*) to measure the amount of uncertainty that the genetic machinery has in relation to the correct decoding of the message transmitted into the nucleus by a signaling pathway.

4) From the value of *H *we can define a function *I *[1] that measures the amount of information content in the input message that the cell's genetic machinery is processing during a given time interval.

5) Combining the information theory with the frequency response analysis of dynamical systems we can examine the cell's genetic response to sinusoidal input signals with varying frequencies and determine if the cell can distinguish between different regimes of information flow from the environment.

6) In the particular case of the ethylene signaling pathway the amount of information managed by the root cell can be correlated with the frequency of the input signal. The ethylene signaling pathway cuts off very low and very high frequencies, allowing a window of frequency response in which the nucleus reads the incoming message as a sinusoidal input. Out of this window the nucleus reads the input message as an approximately non-varying one.

7) This frequency response analysis is also useful to estimate: a) the gain of the system during the synthesis of the protein ERF1 (~-5.6 dB); b) the rate of information transfer (~3 millibits) during the transport of each new ERF1 molecule into the nucleus and c) the time of synthesis of each new ERF1 molecule (~21.3 s).

8) In the case of the system of a single master gene (*ERF*1) and a single slave gene (*HLS*1), the total Shannon entropy is completely determined by the uncertainty associated with the expression of the master gene.

9) The Shannon entropy associated with the expression of the *HLS*1 gene determines the information content of the system that is related to its interaction with the antagonistic genes *ARF*1, 2.

## Authors' contributions

Both authors have made equal substantive contributions to this manuscript. Both authors have read and approved the final manuscript.

## Supplementary Material

Additional File 1 **Supplementary Material**. Full mathematical model of the activation of the ethylene-signaling pathway. Click here for file

Additional File 2 **Table 2**. One-cell ethylene model: differential equations and parameters. Click here for file
