# Supplementary material for: Information flow during gene activation by signaling molecules: ethylene transduction in Arabidopsis cells as a study system
Source: BMC Syst Biol. 2009 May 5;3:48. doi: 10.1186/1752-0509-3-48 (PMC2688479; doi:10.1186/1752-0509-3-48)
Supplement: Additional File 1 — Supplementary Material. Full mathematical model of the activation of the ethylene-signaling pathway. [file 1752-0509-3-48-S1.pdf]

Supplementary Material

Activation of the MAPK module

In the model, we assume that the total concentration of ethylene receptors in the ER membrane (denoted by etr_T) is ~ 0.3 μM. The activated state of the receptor is denoted by $etr^{(+)}$ and its inactivated state by $etr^{(-)}$. In the absence of ethylene, all receptors are in their constitutive activated state, and $etr^{(+)} = etr_T$. In the presence of ethylene, both states obey the balance equation:

$$etr^{(+)} + etr^{(-)} = etr_T \qquad [1]$$

In this form, if we use *ctr1** to denote the concentration of the activated constitutive triple response 1 molecule and *mapkk** and *mapk** to denote the activated state of the kinases downstream of *ctr*1*, we obtain the following balance equations for the activation rates of the kinases that conform the MAPK module at the ER lumen of the root cell:

$$\frac{dctr1^*}{dt} = \kappa_3\left(etr_T - etr^{(-)}\right)\left(ctr_T - ctr1^*\right) - \kappa_4 ctr1^* \qquad [2]$$

$$\frac{dmapkk^*}{dt} = \kappa_5 ctr1^*\left(mapkk_T - mapkk^*\right) - \kappa_6 mapkk^* \qquad [3]$$

$$\frac{dmapk^*}{dt} = \kappa_7 mapkk^*\left(mapk_T - mapk^*\right) - \kappa_8 mapk^* \qquad [4]$$

Equation [2] indicates that the velocity of activation of CTR1 is directly proportional to the product of the concentration of activated ETR and to the amount of inactivated CTR1 molecules minus the rate of deactivation of the activated CTR1 molecule.

We assume that one activated CTR1 molecule is associated at each and every moment to the activated ETR; thus, $ctr1_T$ ~ 0.3 μM in our model. The value of the κ_3 rate constant of Eqn. [2] was adjusted by assuming that at zero ethylene concentration all of the CTR1* molecules are in a steady activated state. The value of κ_4 was based on the observations of Chen and Bleecker (1995) that at very low values of ethylene concentration (<<0.001 μL/L) almost all of the CTR1 molecules are in their steady activated state. The values of these parameters for *CTR1** ~ 0.29 μM for low ethylene concentrations are given in **Table 2, main text.**

The activation of the kinases downstream of CTR1* are modeled following Yamada, Taketomi and Yoshimura (2004) and Diaz and Martinez-Mekler (1995), giving rise to Eqns. [3] and [4]. In these equations, $MAPKK_T$ and $MAPK_T$ are assumed to be ~0.5 μM (7), which is equivalent to a total amount of ~2,370 molecules of each kinase in the ER. Equation [3] indicates that the rate of activation of the MAPKK is a balance between its rate of activation by CTR1* and its rate of inactivation by its phosphatase. Equation [4] indicates that the rate of activation of the MAPK is a balance between its rate of activation by MAPKK* and its rate of inactivation by its phosphatase.

The values of the rate constants of Eqns. [3] and [4] are taken from Yamada et al. (2004), assuming, as a first approximation, that the kinetics of activation of the MAPKK and MAPK molecules in the *Arabidopsis* cells is very similar to that observed for MEK and ERK in animal cells (**see Table 2 in main text**).

Inactivation of EIN2

MAPK* blocks the EIN2 molecule of the nuclear membrane at a rate given by:

$$\frac{dein2^{(-)}}{dt} = \kappa_9 mapk_c * \left(ein2_T - ein2^{(-)}\right) - \kappa_{10} ein2^{(-)} \frac{etr^{(-)}}{etr^{(-)} + \beta} \quad [5]$$

where $ein2^{(-)}$ represents the concentration of inactivated ein2 in the nucleus and $ein2_T$ its total concentration. According to the above equation, the rate of inactivation of EIN2 is a balance between its rate of phosphorylation by MAPK* and its rate of dephosphorylation by a still unknown process. However, we assume that it depends on the rate of inactivation of the ethylene receptors according to a Michaelis-Menten behavior.

In this equation, we assume that the total concentration of EIN2 (denoted by $ein2_T$) is 0.005 μM with respect to the nucleus, which is equivalent to ~1,580 molecules of the channel. In the absence of ethylene, all of the EIN2 molecules are in their phosphorylated state; thus, $ein2^{(-)} = ein2_T$. We then adjust the rate constants in order to obtain $ein2^{(-)} \approx ein2_T \approx 0.005$ μM for very low concentrations of ethylene (<<0.001 μL/L) because the response of the system to ethylene is minimal at these concentrations (Chen & Bleecker, 1995). For other ethylene concentrations, the number of inactivated EIN2 molecules slowly decreases according to the balance equation, $ein2^{(-)} + ein2^{(+)} = ein2_T$. Consequently, at high concentrations of ethylene (>10 μL/L), all of the EIN2 molecules are in their dephosphorylated state and $ein2^{(+)} = ein2_T$. The respective values of the rate constants of Eqn. [5] are shown in **Table 2, main text**.

*mapkc** represents the corrected concentration of the activated MAPK with respect to the nuclear volume in Eqn. [5]. The form in which this concentration is scaled is shown in the **Table 2, Section 5, main text**.

Inactivation of the MAPK cascade and activation of the ethylene response

Inactivation of the MAPK cascade occurs when the ethylene gas binds to either of its specific receptors at a rate proportional to the amount of ethylene gas still unbound and to the number of receptor molecules that are still in their activated state. Thus, the rate of inactivation of the receptor is given by:

$$\frac{\det r^{(-)}}{dt} = \kappa_1 (ET - etr^{(-)})\left(etr_T - etr^{(-)}\right) - \kappa_2 etr^{(-)} \qquad [6]$$

where *ET* represents the total amount of ethylene gas, which is a control parameter. This equation is subject to balance equation [1]. The values of the rate constants of Eqn. [6] were obtained from the work of Chen and Bleecker (1995) in that at very high concentrations of ethylene (>10 μL/L), the MAPK cascade should be completely inactivated; thus, $etr^{(-)}$ ~ 0.3 μM. These values are shown in the **Table 2, main text**.

Once the MAPK has been blocked by the presence of ethylene, the EIN2 molecule of the nuclear membrane is activated at a rate given by:

$$\frac{dein2^{(+)}}{dt} = -\frac{dein2^{(-)}}{dt} \qquad [7]$$

subject to $ein2^{(-)}$ + $ein2^{(+)}$ = $ein2_T$ (see Eq. [5] above).

Once EIN2 has been activated, the EIN3 molecule inside the nucleus is activated by a still unknown process at a rate given by:

$$\frac{dein3^*}{dt} = \kappa_{11} ein2^{(+)} \left(ein3_T - ein3^* \right) - \kappa_{12} ein3^* \quad [8]$$

where $ein3^*$ is the concentration of the activated form of the EIN3 transcription factor in the nucleus, and $ein3_T$ is the total concentration of the molecule in the nucleus. We assume that $ein3_T$ = 0.005 μM, which is equivalent to ~1,580 molecules, which has been taken to be the size of the pool of this molecule in the nucleus. The rate constants in Eqn. [8] were adjusted in order to obtain a value of EIN3* ~ 0.005 μM for high concentrations of ethylene. (**See Table 2, Section 7 in main text**).

The activated EIN3 transcription factor binds to the promoter site of the ***ERF1*** gene, thereby allowing its expression. Assuming that the probability of activation of ***ERF1*** by EIN3 depends only of the gene's promoter site state (*occupied by* EIN3 *or empty*) at time *t*, we can establish that the rate at which the probability of the activated state (or state *on*) of the gene ***ERF1*** changes in a given time interval $t + \Delta t$ is a balance between the rate at which the gene changes from its state *off* to its state *on* and the rate at which the gene changes from its state *on* to its state *off*. The first term of this balance depends on the number of activated EIN3 transcription factor molecules in the nucleus at time *t* and on the

probability of the gene being in its state *off* at time *t*. The second term in the balance equation depends only on the probability that the gene is in its state *on* at time *t*. As: $\frac{dp_{ERF1}^{off}(t)}{dt} = -\frac{dp_{ERF1}^{on}(t)}{dt}$ the two-state Markov model for this process is given by the equations:

$$\frac{dp_{ERF1}^{on}(t)}{dt} = \kappa_{13} N_{EIN3*} p_{ERF1}^{off}(t) - \kappa_{14} p_{ERF1}^{on}(t)$$

$$\frac{dp_{ERF1}^{off}(t)}{dt} = -\kappa_{13} N_{EIN3^*} p_{ERF1}^{off}(t) + \kappa_{14} p_{ERF1}^{on}(t) \qquad [9]$$

where the rate constants were chosen from a set of values for which $p_{ERF1}^{on} > 0.95$ when the concentration of ethylene is greater than 10 μL/L (see Table 2, Section 7, main text). In the absence of ethylene, the *ERF1* gene is in the *off* state with a probability 1. The number of activated EIN3* molecules for these equations can be easily calculated as:

$$N_{EIN3*} = \left(1x10^{-21}\right) EIN3 * V_{nucleus} N_{Avogadro} \qquad [10]$$

Activation of target genes downstream *ERF1*

Once the ***ERF1*** gene has been activated in response to ethylene, it is transcribed, as a first approximation, at a rate given by:

$$\frac{dmRNA}{dt} = \frac{p_{ERF1}^{on}(t) V_{trans} mRNA}{mRNA + \kappa_{15}} - \kappa_{16} mRNA \qquad [11]$$

where *mRNA* is the nuclear concentration of the transcripts of the ***ERF1*** gene, and V_{trans} is the maximum transcription rate of ***EFR1*** (**see Table 2, Section 7, main text**), which is obtained when p^{on}_{ERF1} = 1. According to Goutsias and Kim (2004), the transcription rate for mRNA is of the order of 0.0001 pM s^{-1} to 1 pM s^{-1}. We used a slightly higher value of 0.000003 μM s^{-1} (3 pM s^{-1}) in order to obtain a steady pool of a rounded-off number of mRNA molecules in the nucleus (~1,000); this value is also obtained when the values reported in the Table 2, Section 7, for the other rate constants of Eqn. [11] are taken into consideration.

The rate of production of the ERF1 protein is then:

$$\frac{derf1}{dt}\Big|_{ER} = \kappa_{17} mRNA - D_{erf1} erf1 \qquad [12]$$

where k_{17} is the translation rate and $D_{erf1}erf1$ is the amount of ERF1 protein moved (by some yet unknown mechanism) from the ER into the nucleus. According to Goutsias and Kim (2004), k_{17} has an estimated value between 0.05 s^{-1} and 0.20 s^{-1}. In our model, we considered a value of 0.1972 s^{-1} (see Table 2, Section 7), for which the number of ERF1 molecules in ER is also ~1,000 molecules at an ethylene concentration of greater than 10 μL/L.

In the nucleus and following an adjustment of the corresponding concentration (see **Table 2, Section 5, main text**), the amount of ERF1 varies according to the balance equation:

$$\frac{derf1}{dt}\Big|_{nucleus} = D_{erf1}erf1n - \kappa_{18}erf1n \qquad [13]$$

where the first term of the right side of Eqn. [13] represents the amount of ERF1 moved from the ER into the nucleus (*ERF1n*), and the second term represents the degradation of the protein. In the model, we assigned the value of 0.99 s^{-1} to D_{erf1}; at this value, most of the ERF1 molecules produced in the ER are moved into the nucleus. k_{18} is then adjusted to obtain a steady concentration of ~1,000 ERF1 molecules for high concentrations of ethylene (see **Table 2, Section 7**).

Finally, we can then model, as a first approximation, the activation of the ***PDF1*** gene with a two-state Markov model (see ***ERF1*** activation Markov model) given by:

$$\begin{aligned}\frac{dp^{on}_{PDF1}(t)}{dt} &= \kappa_{19}N_{ERF1n}p^{off}_{PDF1}(t) - \kappa_{20}p^{on}_{PDF1}(t) \\ \frac{dp^{off}_{PDF1}(t)}{dt} &= -\kappa_{19}N_{ERF1n}p^{off}_{PDF1}(t) + \kappa_{20}p^{on}_{PDF1}(t)\end{aligned} \qquad [14]$$

The inactivation of the ***HLS1*** gene is given by another two-state Markov model (see ***ERF1*** activation Markov model):

$$\frac{dp_{HLS1}^{on}(t)}{dt} = \kappa_{21} N_{ERF1n} p_{HLS1}^{off}(t) - \kappa_{22} p_{HLS1}^{on}(t)$$

$$\frac{dp_{HLS1}^{off}(t)}{dt} = -\kappa_{21} N_{ERF1n} p_{HLS1}^{off}(t) + \kappa_{22} p_{HLS1}^{on}(t) \qquad [15]$$

where N_{ERF1n} is calculated as indicated in the Table 2, Section 4 in the main article.

With respect to Eqns. [14] and [15], the value of the rate constants were adjusted to obtain a p^{on} > 0.75 for high ethylene concentrations (>10 μL/L) (see **Table 2, Section 7**).

As we do not know the posttranslational mechanism by which the expression of the gene ***ARF2*** is negatively regulated by the ***HLS1*** gene (Li et al., 2004) we modeled the expression of ***ARF2*** as a three-state discrete variable given by:

$$p_{ARF2}^{on}(t) = \begin{cases} 0 & \text{if } p_{HLS1}^{on}(t) > 0.75 \\ 0.5 & \text{if } 0.5 < p_{HLS1}^{on}(t) \leq 0.75 \\ 1 & \text{if } p_{HLS1}^{on}(t) \leq 0.5 \end{cases} \qquad [16]$$

Thus, according to Eqn. [16], if ***HLS1*** is fully expressed at time *t*, the probability of expression of ***ARF2*** is 0 at time *t*. In this form, we are combining a continuous approach (***HLS1*** activation) with a three-state, time-dependent discrete approach (***ARF2*** negative regulation).

If we remove Eqns. [3] and [4] from the set of equations, the dynamical features of the model do not change, indicating that the role of the kinases downstream of CTR1, if they exist, is only to transmit the signal from the ETR to EIN2.
