# Supplementary material for: Information flow during gene activation by signaling molecules: ethylene transduction in Arabidopsis cells as a study system
Source: BMC Syst Biol. 2009 May 5;3:48. doi: 10.1186/1752-0509-3-48 (PMC2688479; doi:10.1186/1752-0509-3-48)
Supplement: Additional File 2 — Table 2. One-cell ethylene model: differential equations and parameters. [file 1752-0509-3-48-S2.pdf]

Table 2.- One-cell ethylene model: differential equations and parameters (ET is given in μM).

<table>
<tr>
<td>

1. Equations describing inactivation of the ethylene receptor:

$$\frac{detr^{(-)}}{dt} = \kappa_1 (ET - etr^{(-)})(etr_T - etr^{(-)}) - \kappa_2 etr^{(-)}$$

$$etr_T = etr^{(+)} + etr^{(-)}$$

</td>
<td>

2. Equations describing the CTR1 module:

$$\frac{dctr1^*}{dt} = \kappa_3 (etr_T - etr^{(-)})(ctr1_T - ctr1^*) - \kappa_4 ctr1^*$$

$$\frac{dmapkk^*}{dt} = \kappa_5 ctr1^* (mapkk_T - mapkk^*) - \kappa_6 mapkk^*$$

$$\frac{dmapk^*}{dt} = \kappa_7 mapkk^* (mapk_T - mapk^*) - \kappa_8 mapk^*$$

</td>
</tr>
<tr>
<td>

3. Equations describing the EIN2 module:

$$\frac{dein2^{(-)}}{dt} = \kappa_9 mapkc^* (ein2_T - ein2^{(-)}) - \kappa_{10} ein2^{(-)} \frac{etr^{(-)}}{etr^{(-)} + \beta}$$

$$ein2_T = ein2^{(+)} + ein2^{(-)}$$

$$\frac{dein2^{(+)}}{dt} = -\frac{dein2^{(-)}}{dt}$$

$$\frac{dein3^*}{dt} = \kappa_{11} ein2^{(+)} (ein3_T - ein3^*) - \kappa_{12} ein3^*$$

$$\frac{dmRNA}{dt} = \frac{p^{on}(t) V_{trans} mRNA}{mRNA + \kappa_{15}} - \kappa_{16} mRNA$$

$$\left.\frac{derf1}{dt}\right|_{ER} = \kappa_{17} mRNA - D_{erf1} erf1$$

$$\left.\frac{derf1}{dt}\right|_{nucleus} = D_{erf1} erf1n - \kappa_{18} erf1n$$

</td>
<td>

4. Equations describing the activation of *ERF1*, *PDF1*, and *ARF2* genes:

$$\frac{dp^{off}_{ERF1}}{dt} = -\kappa_{13} N_{ein3} p^{off}_{ERF1} + \kappa_{14} p^{on}_{ERF1}$$

$$\frac{dp^{on}_{ERF1}}{dt} = \kappa_{13} N_{ein3} p^{off}_{ERF1} - \kappa_{14} p^{on}_{ERF1}$$

$$N_{ein3} = 602.3 V_{nucleus} EIN3^*$$

$$\frac{dp^{on}_{PDF1}(t)}{dt} = \kappa_{19} N_{ERF1n} p^{off}_{PDF1}(t) - \kappa_{20} p^{on}_{PDF1}(t)$$

$$\frac{dp^{off}_{PDF1}(t)}{dt} = -\kappa_{19} N_{ERF1n} p^{off}_{PDF1}(t) + \kappa_{20} p^{on}_{PDF1}(t)$$

$$\frac{dp^{on}_{HLS1}(t)}{dt} = \kappa_{21} N_{ERF1n} p^{off}_{HLS1}(t) - \kappa_{22} p^{on}_{HLS1}(t)$$

$$\frac{dp^{off}_{HLS1}(t)}{dt} = -\kappa_{21} N_{ERF1n} p^{off}_{HLS1}(t) + \kappa_{22} p^{on}_{HLS1}(t)$$

$$N_{ERF1n} = 602.3 V_{nucleus} ERF1$$

</td>
</tr>
<tr>
<td>

5.- Adjustment of the MAPK concentration from ER to nucleus:

$$n_{MAPK} = 5x10^{-21} mapk^*$$

$$mapkc^* = 1.908x10^{18} n_{MAPK}$$

Adjustment of ERF1 concentration from ER to nucleus:

$$n_{erf1} = 5x10^{-21} erf1$$

$$erf1n = 1.908x10^{18} n_{erf1}$$

</td>
<td>

6.- Initial conditions:

$ctr1^*(0) = 0.3\mu M$ $erf1(0) = 0;$ $p^{on}_{ERF1}(0) = 0;$

$etr^{(-)}(0) = 0$ $erf1n(0) = 0;$ $p^{off}_{ERF1}(0) = 1;$

$mapkk^*(0) = 0.5\mu M;$ $p^{off}_{PDF1}(0) = 1;$

$mapk^*(0) = 0.5\mu M;$ $p^{on}_{PDF1}(0) = 0;$

$ein2^{(-)}(0) = 0.005\mu M;$ $p^{off}_{HLS1}(0) = 1;$

$ein3^*(0) = 0;$ $p^{on}_{HLS1}(0) = 0;$

</td>
</tr>
<tr>
<td>

7. Parameter values:

$\kappa_1 = 5\mu M^{-1} s^{-1}$; $\kappa_2 = 0.0003 s^{-1}$; $\kappa_3 = 3\mu M^{-1} s^{-1}$;
$\kappa_4 = 0.085 s^{-1}$; $\kappa_5 = 9.196\mu M^{-1} s^{-1}$; $\kappa_6 = 4.598 s^{-1}$; $\kappa_7 = 0.318\mu M^{-1} s^{-1}$;
$\kappa_8 = 0.0954 s^{-1}$; $\kappa_9 = 2\mu M^{-1} s^{-1}$; $\kappa_{10} = 0.005 s^{-1}$;
$\kappa_{11} = 5\mu M^{-1} s^{-1}$;
$\kappa_{12} = 0.005 s^{-1}$; $\kappa_{13} = 0.003 s^{-1}$; $\kappa_{14} = 0.09 s^{-1}$; $\kappa_{15} = 0.0001\mu M$;
$\kappa_{16} = 0.0009 s^{-1}$; $\kappa_{17} = 0.1972 s^{-1}$; $\kappa_{18} = 0.198 s^{-1}$; $\kappa_{19} = 0.0025 s^{-1}$;
$\kappa_{20} = 0.61 s^{-1}$; $\kappa_{21} = 0.003 s^{-1}$; $\kappa_{22} = 0.65 s^{-1}$; $\beta = 6\mu M$;
$V_{trans} = 0.000003\mu M s^{-1}$; $D_{erf1} = 0.99 s^{-1}$; $etr_T = 0.3\mu M$;
$ctr1_T = 0.3\mu M$; $mapkk_T = 0.5\mu M$; $mapk_T = 0.5\ \mu M$;
$ein2_T = 0.005\mu M$; $ein3_T = 0.005\mu M$; $V_{nucleus} = 524\mu m^3$.

</td>
<td>

8. Variables:
etr = concentration of ethylene receptor at the cell membrane; *ET* = total concentration of ethylene outside the cell (**control variable**); *ctr1** = concentration of activated constitutive triple response1 protein; $mapkk^*$ = concentration of activated mitogen-activated protein kinase kinase; $mapk^*$ = concentration of activated mitogen-activated protein kinase; *ein2* = concentration of ethylene-insensitive protein 2; *ein3** = concentration of activated ethylene-insensitive protein 2; *mRNA* = concentration of messenger RNA in nucleus; *erf1* = concentration of ERF1 transcription factor; N_{ein3} = number of activated EIN3 molecules in the nucleus; N_{erf1} = number of activated ERF1 molecules in the nucleus; $V_{nucleus}$ = nuclear volume; n_{MAPK} = number of moles of activated MAPK; $mapkc^*$ = concentration of MAPK in the nucleus; n_{erf1} = number of moles of activated ERF1 molecules; *erf1n* = concentration of activated ERF1 transcription factor in the nucleus. The symbol **(+)** means the activated form of the corresponding receptor and EIN2 molecule, while the symbol **(-)** represents their inactivated form. The subscript *T* stands for the total concentration of the corresponding molecule. The superscript * means the active state of the molecule.

</td>
</tr>
</table>
